# Atraumatic Intercostal and Intrathoracic Liver Herniation Related to Influenza A

**DOI:** 10.14309/crj.0000000000000427

**Published:** 2020-07-23

**Authors:** Prateek S. Harne, Samiran Mukherjee, Zachary Shepherd

**Affiliations:** 1 SUNY Upstate Medical University, Syracuse, New York

## Abstract

Nontraumatic and spontaneous intercostal and intrathoracic herniations are defined as protrusions of intra-abdominal contents through acquired or congenital defects of the abdominal and thoracic walls without any proceeding trauma and are sparsely reported in the literature with less than 35 detailed case reports reported in the literature worldwide. Most of these cases result from abdominal trauma and are considered surgical emergencies. The content of these herniations, as reported in the literature, have classically been lungs and intra-abdominal organs. We report a case of nontraumatic intercostal and intrathoracic liver herniation, which was managed conservatively given minimal liver injury and rapidly improving symptoms.

## INTRODUCTION

Nontraumatic and spontaneous intercostal and intrathoracic herniations are sparsely and separately reported in the literature. They are defined as a protrusion of intra-abdominal contents through acquired or congenital defects of the abdominal and thoracic walls. A nontraumatic adult liver herniation is sparsely reported with less than 35 case reports on a review of literature worldwide since 1956.^1,2^ Most cases of thoracoabdominal hernia are commonly a result of blunt or penetrating trauma.^3^ There have been individual cases reporting such herniation of the lungs, small and large bowel, omentum, and liver.^4^ We describe a patient who had both intercostal and diaphragmatic liver herniation secondary to increased cough, which to our knowledge, based on PubMed, Cochrane review, and Google Scholar review, is a very rare case of nontraumatic intercostal and intrathoracic liver herniation. The literature relies on computed tomography for diagnosis and for assessing damage to liver parenchyma, which guides management. Although intercostal liver herniations can be surgical emergencies, our case was managed conservatively.

## CASE REPORT

**Figure 1.**
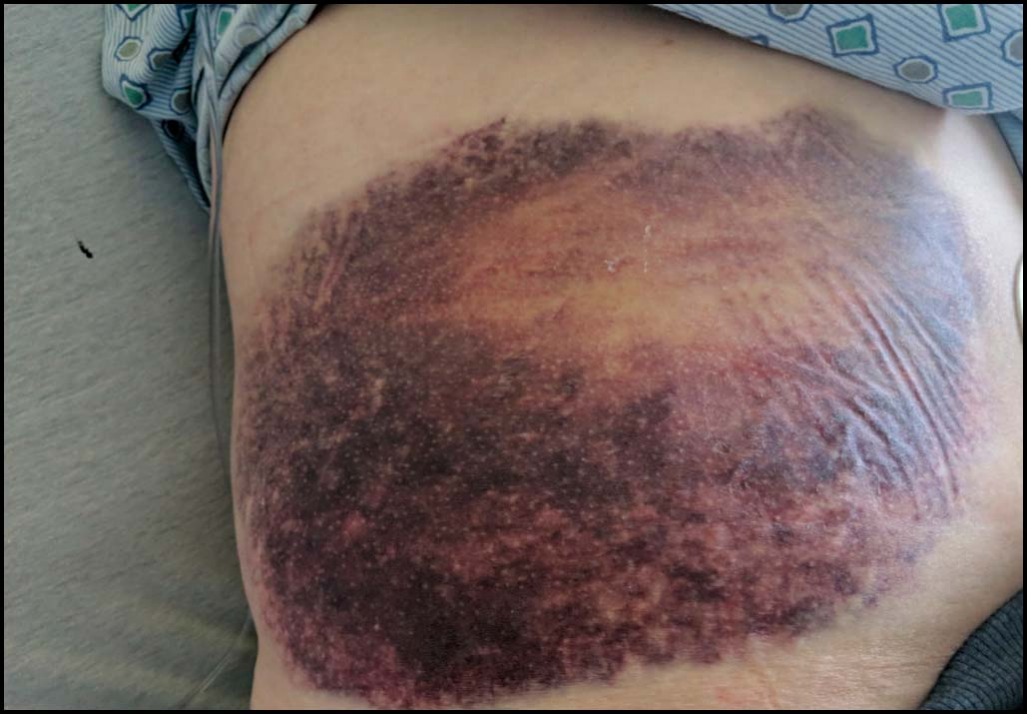
Image showing ecchymoses measuring 30 × 25 cm which was present on admission that gradually increased from 5 × 5 cm. There was noticeable swelling of this entire area, and it was tender to palpation.

A 58-year-old woman with a medical history of hypothyroidism and essential hypertension presented with severe dry cough and nasal congestion for 2 weeks. Three days before presentation, she had noticed a bruise on her right lateral chest wall which gradually increased in size and became painful with radiation to her back. The patient denied any recent history of trauma to the area. Her review of systems was positive for the presence of dry cough. Physical examination revealed a bulging, palpable, and tender ecchymotic lesion over the right ninth and 10th ribs in addition to clear lungs (Figure 1). Vitals were unremarkable. Laboratory test results were significant for alanine aminotransferase level of 41 U/L, aspartate aminotransferase level of 62 U/L, creatinine kinase level of 493 U/L, white blood cell count of 17,800 cells/uL, platelet count of 567,000 cells/uL, international normalized ratio 0.9, and a positive nasal swab for influenza A.

**Figure 2.**
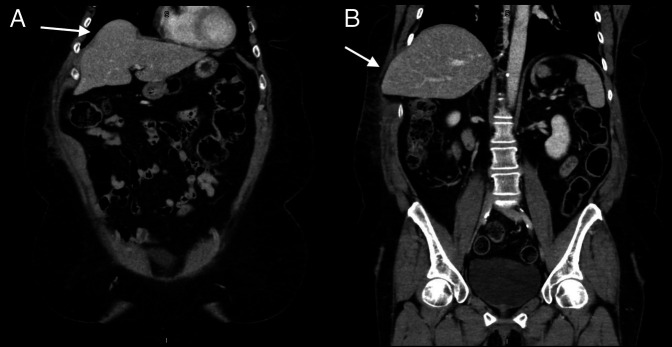
(A) Abdominal computed tomography without contrast showing herniation of liver intro the intrathoracic cavity (or the “hump sign”). (B) Abdominal and pelvic computed tomography demonstrating herniation of an uninjured liver between the ninth and 10th intercostal space to impinge on the subcutaneous tissue.

Abdominal computed tomography showed herniation of the uninjured liver through a diaphragmatic defect into the thoracic cavity (“hump sign”) with a concomitant intercostal herniation between ninth and 10th ribs (Figure 2). Also reported was the presence of abdominal wall soft tissue stranding (or edema). Surgical services were consulted, and the patient was managed conservatively with opiate analgesia, intravenous hydration with normal saline, cough suppression, and Oseltamivir. She was then discharged with a close medical and surgical follow-up. In the 1- and 6-month follow-ups, the patient had minimal symptoms in the form of abdominal contour change, with the resolution of ecchymosis and tenderness to palpation, which was previously present. Laboratory test results had returned to reference range on the follow-up visit.

## DISCUSSION

Croce and Mehta first coined intercostopleuropertioneal hernia, whereas Cole et al first reported transdiaphragmatic intercostal hernia which resulted from violent coughing spells.^5,6^ Based on a review of PubMed, Cochrane review, and Google Scholar, this seems to be a rare case of nontraumatic, spontaneous intrathoracic, and intercostal liver herniation. There have been cases reporting both these herniations separately as discussed below. Individual reports have linked sudden increase in thoracoabdominal pressure gradient such as violent coughing spell, heavy lifting, and parturition as a predisposing risk factor for herniation, with coughing being the most common of them all.^1–4,7–9^ Commonly, the symptoms of spontaneous intercostal herniation are thoracoabdominal pain, nausea, and vomiting, which are similar to intrathoracic herniation, where dyspnea may also be present and all of these symptoms were present in our patient.^1,2,9,10^ Ecchymosis or bulge or both over the hernia is usually seen in patients with concomitant intercostal muscle rupture as was seen in our patient.^2,10^

Macedo et al reported 3 cases of transdiaphragmatic intercostal hernia secondary to cough.^11^ Contents for 2 of these were intestinal loops, and 1 of them was liver, and all of them were treated with thoracotomy. A review of 20 cases of spontaneous diaphragmatic rupture of diaphragm (variable intraabdominal hernial contents) by Losanoff et al were also repaired by thoracotomy as compared to our case, which was managed conservatively.^1^ A case reported by Bendinelli et al, commented on the failure of conservative measures for a lacerated liver herniation, which led to liver necrosis, ultimately needing surgery.^7^

Surgical interventions in our case were deferred because there was no evidence of laceration and instead, a close follow-up was implemented. A key derivation from this review is to maintain a high suspicion of liver herniation in patients with poor abdominal muscle strength with sudden worsening of cough. The herniation may either be abdominal or transdiaphragmatic, both of which need evaluation. Given the rarity of such events, these patients can present as a diagnostic challenge, and delay in care can result in poor outcomes, especially in patients with organ damage. A consensus for management can be difficult because of the sparsely reported cases. Although an injured or strangulated liver herniation is a surgical emergency, as has been reported in the literature, we propose the possibility of a conservative approach for uninjured liver herniation. This would also include close medical and surgical follow-up after discharge from the hospital.

## DISCLOSURES

Author contributions: All authors contributed equally to this manuscript. PS Harne is the article guarantor.

Financial disclosure: None to report.

Previous presentation: This case was presented at the American College of Gastroenterology Annual Scientific Meeting; October 25-30, 2019; San Antonio, Texas.

Informed consent was obtained for this case report.
